# The twin-beginnings of COVID-19 in Asia and Europe—one prevails quickly

**DOI:** 10.1093/nsr/nwab223

**Published:** 2021-12-11

**Authors:** Yongsen Ruan, Haijun Wen, Mei Hou, Ziwen He, Xuemei Lu, Yongbiao Xue, Xionglei He, Ya-Ping Zhang, Chung-I Wu

**Affiliations:** State Key Laboratory of Biocontrol, School of Life Sciences, Southern Marine Science and Engineering Guangdong Laboratory (Zhuhai), Sun Yat-sen University, Guangzhou510275, China; State Key Laboratory of Biocontrol, School of Life Sciences, Southern Marine Science and Engineering Guangdong Laboratory (Zhuhai), Sun Yat-sen University, Guangzhou510275, China; State Key Laboratory of Biocontrol, School of Life Sciences, Southern Marine Science and Engineering Guangdong Laboratory (Zhuhai), Sun Yat-sen University, Guangzhou510275, China; State Key Laboratory of Biocontrol, School of Life Sciences, Southern Marine Science and Engineering Guangdong Laboratory (Zhuhai), Sun Yat-sen University, Guangzhou510275, China; State Key Laboratory of Genetic Resources and Evolution, Kunming Institute of Zoology, Chinese Academy of Sciences, Kunming650223, China; Beijing Institute of Genomics, Chinese Academy of Sciences, and China National Centre for Bioinformation, Beijing100101, China; State Key Laboratory of Biocontrol, School of Life Sciences, Southern Marine Science and Engineering Guangdong Laboratory (Zhuhai), Sun Yat-sen University, Guangzhou510275, China; State Key Laboratory of Genetic Resources and Evolution, Kunming Institute of Zoology, Chinese Academy of Sciences, Kunming650223, China; State Key Laboratory of Biocontrol, School of Life Sciences, Southern Marine Science and Engineering Guangdong Laboratory (Zhuhai), Sun Yat-sen University, Guangzhou510275, China; Beijing Institute of Genomics, Chinese Academy of Sciences, and China National Centre for Bioinformation, Beijing100101, China; Department of Ecology and Evolution, University of Chicago, Chicago, IL60637, USA

**Keywords:** SARS-CoV-2, COVID-19, D614G, twin-beginnings, fitness advantage

## Abstract

In the spread of SARS-CoV-2, there have been multiple waves of replacement between strains, each of which having a distinct set of mutations. The first wave is a group of four mutations (C241T, C3037T, C14408T and A23403G [this being the amino acid change D614G]; all designated 0 to 1 below). This DG (D614G) group, fixed at the start of the pandemic, is the foundation of all subsequent waves of strains. Curiously, the DG group is absent in early Asian samples but present (and likely common) in Europe from the beginning. European data show that the high fitness of DG1111 requires the synergistic effect of all four mutations. However, the European strains would have had no time to evolve the four DG mutations (0 to 1), had they come directly from the early Asian DG0000 strain. Very likely, the European DG1111 strain had acquired the highly adaptive DG mutations in pre-pandemic Europe and had been spreading in parallel with the Asian strains. Two recent reports further support this twin-beginning interpretation. There was a period of two-way spread between Asia and Europe but, by May 2020, the European strains had supplanted the Asian strains globally. This large-scale replacement of one set of mutations for another has since been replayed many times as COVID-19 progresses.

## INTRODUCTION

The study of molecular evolution is constrained by the data on extant organisms only. In contrast, the large number of genome sequences of SARS-CoV-2, collected throughout the entire period of the epidemics, has provided an unprecedented opportunity to observe evolution in action [1–6]. While this large data set can be used to track the evolution of SARS-CoV-2 for the entire period of the pandemic, this study will focus on its beginning in early 2020. We choose the extensive UK data to represent Europe in a comparison between Asia and Europe. The patterns in other regions, for example, North America and India, are comparable.

## RESULTS

### The succession of waves of variants in COVID-19

**Figure 1.**
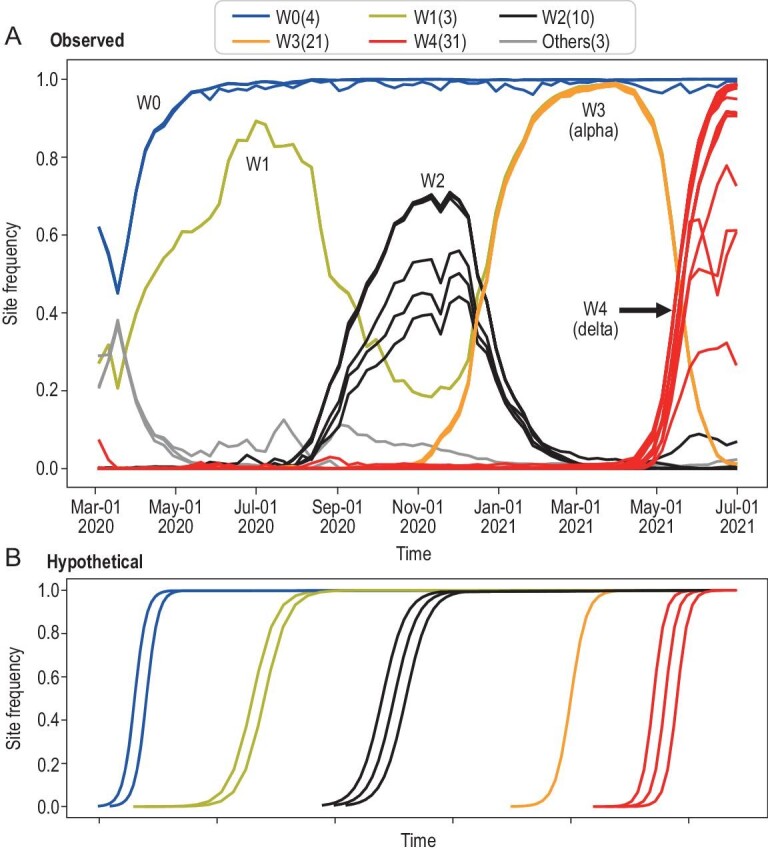
The observed vs. hypothetical evolution of site frequency in SARS-CoV-2. (A) Evolution of SARS-CoV-2 between 1 January 2020 and 1 July 2021 depicted by waves (i.e. successions of ‘mutation groups’) in the UK. Sequencing data were obtained from the GISAID database [35]. The frequency of the mutant at each variable site (e.g. C→ T) is tracked but only variants that reach the frequency cutoff of 0.3 at their peaks are presented. While a curve represents the rise and fall of a variant, each observed curve usually represents multiple curves that overlap completely. In COVID-19, there are five waves (W0 to W4). In W2, the curves do not overlap completely, thus revealing multiple variants of similar dynamics. The numbers of variants [non-synonymous: synonymous: non-coding] for the five waves of W0 to W4 are: 2 : 1 : 1, 2 : 1 : 0, 4 : 5 : 1, 15 : 5 : 1 and 26 : 3 : 2 for W0 to W4, respectively. The total is given in the parentheses next to the wave label above the figure. W3 and W4 correspond to the Alpha and Delta strain while the focus of this study is the W0 wave. W0 has four mutations, collectively referred to as the DG group. (B) The hypothetical site-frequency evolution depicted in the conventional model of molecular evolution whereby mutations are sequentially fixed. Note the contrast between the two panels with only the W0 wave behaving as conventionally portrayed. In both (A) and (B), there are five waves, each of which being composed of a group of mutations portrayed in the same color as they rise (and fall) together as a cluster. In the conventional thinking, waves resemble those of (B) with a series of rises. The observed rises and falls in (A), visually closer to the waves in the physical world, are quite novel in evolution.

The evolutionary dynamics of SARS-CoV-2 in the UK is given in Fig. 1 where the changes in the variant frequency at each site (e.g. C→T) from March 2020 to July 2021 are presented. Briefly, by lining up a large number of viral sequences, we examine each site across sequences using the infinite site model of population genetics [7]. In contrast, virological studies usually examine each sequence across sites [2,4,6,8], akin to using the infinite-allele model of population genetics. Variants are compared with the ancestral state in the outgroup sequences of bats to determine the mutant status. A haplotype is defined as two or more variants of the same viral genome. We filtered out sites where the mutant frequency never reached a cutoff value of 0.1, 0.3 or 0.5. Variants that have never been sufficiently common in the population are of lower interest as they have limited impact on the progression of epidemics. Here, we use 0.3 as the cutoff although the conclusion is the same with the other two cutoffs.

In Fig. 1A, one could observe five waves of variants (labeled W0 to W4; a precise definition of each wave is given in the legend) that rise and fall together in the same wave (data shown in Table S1). Each wave is a composite of multiple overlapping curves with each curve representing a variant at a particular site. The overlap therefore portrays a haplotype that bears multiple variants of the same evolutionary dynamics. Variants of each wave do differ slightly in the low frequency range and, in W2 and W4, the differences can be seen even in higher frequencies. These variants do follow the same trend in the rise and fall. The difference happens when a new variant emerges in the same haplotype, but after others have reached a modest frequency of, say, 10%. The formation of the waves will be discussed in detail below.

An unexpected and most interesting observation in Fig. 1A is that a new wave usually rises at the expense of the previous wave. This is surprising in the analysis of the site-by-site evolution whereby one might have expected cumulative evolution as shown in Fig. 1B [9]. In this mode of evolution, new mutations are piled on the earlier successful haplotypes, resulting in a series of ascending curves depicted in Fig. 1B. Thus, the rises in Fig. 1A are expected but the falls are not. The overall patterns suggest strong competition and mutual exclusion between different sets of mutations. The competition will later help to explain the evolution of SARS-CoV-2 in the early days.

The only wave that rises and stays at the top is W0. It has four variants (the noncoding site C241T, synonymous site C3037T, two nonsynonymous sites C14408T and A23403G, the latter being the D614G site), which will be referred to collectively as the D614G (or DG) group [10–16]. Since the evolution proceeds from CCCA to TTTG, we designate the ancestral haplotype CCCA as DG0000 and the globally successful haplotype TTTG as DG1111. Depending on the bat species used as the outgroup, the ancestral sequence could be DG0000 or DG0100 (see online supplementary material). Because the analyses and conclusion are not affected by this synonymous site, we use DG0000 as the ancestor. Usually, all four DG mutations are either all present or absent. This tight association, however, may not be true when the mutations are still in low frequency and partial haplotypes such as DG1001 can occasionally be seen.

The unique strength of the DG group is evident in their becoming fixed very quickly (Fig. 1A). Other subsequent waves, W1–W3, all went up and down while the latest W4 (the Delta wave) is too recent to judge [17,18].

### The anatomy of Wave 0

**Figure 2.**
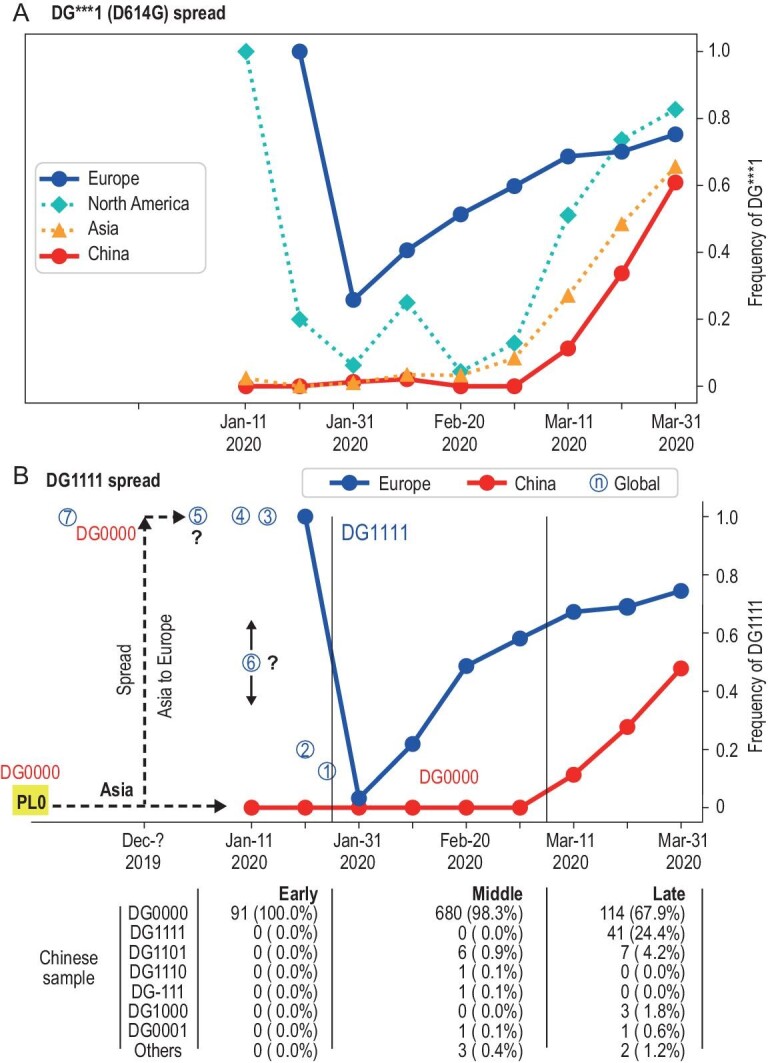
Detailed analyses of the W0 wave before March 2020 under the one-beginning scenario. (A) The frequency of the D614G mutation is tracked among the samples from China, Asia, Europe, and North America. The European samples have higher frequencies of the D614G mutation than the Asian samples at every time point with a drop in February due to the influx of the Asian strains. (B) Frequency change of the DG1111 haplotype (with all four mutations) in China and Europe. The evolution of the DG mutations is divided into three stages: Early (up to 21 January 2020)—all samples in China being DG0000; Middle (21 January–1 March)—a mixture but without DG1111; Late (after 1 March)—DG1111 present in China. While DG1111 is absent in China in the Early stage, it is present, and likely common, in Europe, North America, and Africa as shown by circled numbers. If the one-beginning scenario is correct, DG1111 must have descended from the imported DG0000 of Asia (the black dotted arrow) and would have had less than a month to make the transition. Since the transition should have taken >1 year at a minimum, the one-beginning scenario in untenable. Complete information on partial haplotypes (between DG0000 and DG1111) are given in Table 2. Circled numbers (see Table S6): (1) Australia (1 DG1111 out of 8); (2) Canada (1/5); (3) Sierra Leone (2/2); (4) Japan (Norway type, 1/1); (5) Canada (31/31); (6) Utah, USA (X/14); (7) Italy (Amendola *et al*. 2021). Samples of (5) and (6) show irregularity in data deposition with either sequences or dates removed without explanation.

We shall focus on the first and the only fixed wave, W0, across geographical regions. The numbers of counts of the W0 variants in China and Europe are given in Tables 1 and 2. Figure 2A tracks the frequency change of the D614G mutation in China, Asia, Europe and North America (data shown in Table S2). In the entire period, the D614G mutation frequencies are higher in Europe than in Asia. After 31 January, two forces influence the frequencies of the DG mutations. One is the inflow of the ancestral DG0000 strain from Asia into Europe, thus driving down the frequency of the DG1111 haplotype there. The other one being the fitness advantage that drives up the DG mutation frequency in all regions a few weeks later. Hence, the dynamics in the European and North American samples show a dip due to the import of the Asian strains before gaining in frequency. The Asian trend, in contrast, rose steadily after mid-March with a time-lag behind the European trend. The difference between continents will be informative on the evolution before the epidemics.

**Table 1.** Haplotype frequencies of the D614G group mutations in early samples from China.


Haplotype	Jan-01 2020 (27)	Jan-11 2020 (11)	Jan-21 2020 (53)	Jan-31 2020 (312)	Feb-10 2020 (235)	Feb-20 2020 (80)	Mar-01 2020 (65)	Mar-11 2020 (62)	Mar-21 2020 (83)	Mar-31 2020 (23)

**CCCA (0000)**	27	11	53	306	228	80	65	53	53	8
**TTTG (1111)**	0	0	0	0	0	0	0	7	23	11
**TTCG (1101)**	0	0	0	3	3	0	0	0	4	3
**-TTG (-111)**	0	0	0	1	0	0	0	0	0	0
**TTTA (1110)**	0	0	0	0	1	0	0	0	0	0
**Others**	0	0	0	2	3	0	0	2	3	1

The haplotype frequencies are showed by a 10-day interval. The first period includes all sequences up to 1 January 2020. The total number of sequences are show in parenthesis in the first row.

**Table 2.** Haplotype frequencies of the D614G group mutations in early samples from Europe.


Haplotype	Jan-01 2020 (0)	Jan-11 2020 (0)	Jan-21 2020 (1)	Jan-31 2020 (31)	Feb-10 2020 (32)	Feb-20 2020 (37)	Mar-01 2020 (592)	Mar-11 2020 (4049)	Mar-21 2020 (8323)	Mar-31 2020 (13 677)

**CCCA (0000)**	0	0	0	23	19	18	234	1236	2432	3259
**TTTG (1111)**	0	0	1	1	7 (9)	18	344	2723	5741	10 184
vs. CCCA					0.21	1.00	1.47	2.20	2.36	3.12
*D_w_*					0.54	0.28	0.25	0.17	0.28	AVG ∼ +0.30
**TTCG (1101)**	0	0	0	7	6	1	5 (6)	7	4	7 (11)
vs. CCCA				0.30	0.32		0.024	0.0057		0.0019
*D_w_*				−0.84	−1.03		−0.85	−0.55		AVG ∼ −0.82
**TCTG (1011)**	0	0	0	0	0	0	2	4 (6)	9	11
vs. CCCA								0.0041	0.0037	0.0034
*D_w_*								−0.094	−0.085	AVG ∼ −0.090
**CTTG (0111)**	0	0	0	0	0	0	0	5	11	8
vs. CCCA								0.0040	0.0045	0.0025
*D_w_*								−0.24	−0.59	AVG ∼ −0.42

Table 2 is the counterpart of Table 1 in Europe with the additional information on the relative fitness of different haplotypes. *D_w_* is the fitness advantage of the focus haplotype over DG0000 with the fitness defined by *R* in ${N_t} = {N_0}\ {e^{R \times t}}$. *D_w_* = *R_X_* − *R_Y_* where *X* is for any haplotype *X* and *R_Y_* is for DG0000. AVG is *D_w_* averaged across time periods.

While the trend in Fig. 2A tracks the last one of the four sites (i.e. DG^***^0 vs. DG^***^1 where ^*^ indicates any nucleotide), the evolution of DG1111 involves all four mutations. The fitness of DG1111 has been suggested to be a function of the entire DG group mutations [19]. In this section, we examine the fitness evolution from DG0000 to DG1111. Table 1 shows the haplotype distribution in China, which is uncomplicated. First, the full DG1111 has not evolved in China and its presence from March onward is, on the record, due to the inflow from abroad. Second, in the absence of DG1111, one may look for the partial haplotypes between DG0000 and DG1111. Among the 3-mutant configurations, 6 DG1101, one DG-111 and one DG1110 are found among 783 sequences before the presence of DG1111. Third, there are a few 1- or 2-mutant haplotypes scattered in the background (Table S3).

In contrast, the haplotypes in Europe show a broader distribution. Table 2 shows the number of each haplotype at each given time. We now focus on DG0000, DG1111 and the 3-mutant haplotypes DG 0111, 1011, 1101 and 1110. The occurrences of DG1111 as well as each partial haplotype are compared with that of DG0000. The changes in the relative abundance manifest the fitness differences among haplotypes. Most noteworthy is the abundance of DG1111 vs. DG0000 that rises from a ratio of 0.21 on 10 February 2020, to 1.0, 1.47, 2.2, 2.36 and then arrives at 3.12 on 31 March. We assume that each strain grows in number by ${N_t} = {N_0} {e^{R \times t}}$ where *N_t_* is the number at time *t*. The fitness advantage, *D_w_*, of strain *X* over strain *Y* is then represented by *R_X_* − *R_Y_*. Here, strain *Y* is always DG0000 and strain *X* is DG1111 or a partial haplotype. The calculation of *D_w_* is based on the sample of each time point against the latest Mar-31 sample in Table 2 and is measured over a 10-day interval.

It is most interesting that the only haplotype with a higher fitness than DG0000 is DG1111, which shows *D_w_* ∼ 0.30 for the average. For all 3-mutant configurations, *D_w_* is negative meaning lower proliferation than the wild-type DG0000. In particular, the most common partial haplotype, DG1101, has a *D_w_* ∼ −0.82 on average. Given the estimated *D_w_*, the frequency of DG1111 relative to that of DG0000 over a 3-month period would be *e*^0.30×9 ^= 14.9 whereas DG1101 would be reduced to 0.0006 of the DG0000. The higher fitness of DG1111 over all partial haplotypes suggests some fitness effect for all four mutations. The first of the four mutations is the noncoding site C241T, which is surprisingly important as *D_w_* would change from 0.30 to −0.42 between DG1111 and DG01111. The second mutation is a synonymous site C3037T, which should be the least important one and indeed DG1011 appears to be most fit among the partial haplotypes.

### The formation of a multi-variant haplotype, DG1111

**Figure 3.**
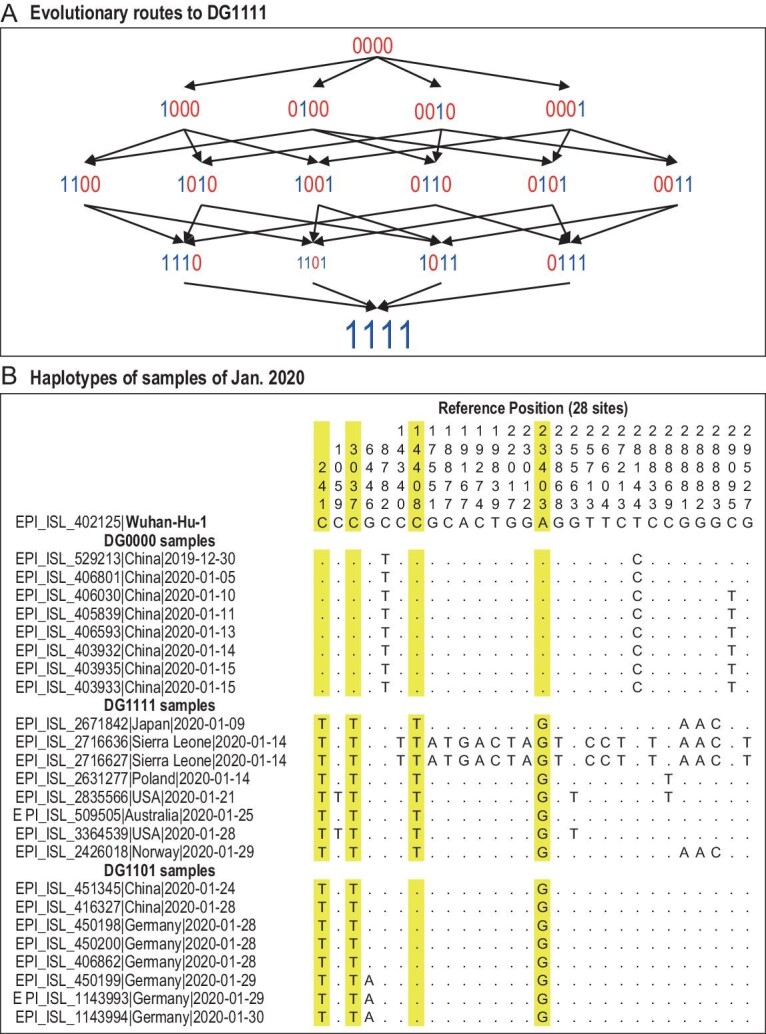
(A) The routes of evolution from DG0000 to DG1111. The route map shows the complexity of evolving a full haplotype. Note the low fitness of the four haplotypes that are one mutation short of DG1111. The fitness is reflected in the font size with DG1111 being the largest and DG1101 being the smallest (see Table 2). Hence, the DG0000 to DG1111 evolution might not have taken place in the current genetic background, thus hinting earlier evolution of DG1111 in an older background than portrayed in Fig. 2. (B) The genomic sequences of the three major haplotypes collected before 30 January 2020. The yellow highlight depicts the four DG group mutations. Note that the youngest haplotype DG1111 is the most diverse. The diversity suggests that DG1111 may have been relatively common in Europe no later than late 2019.

The analysis above provides us with the means to roughly estimate the time it may take for DG0000 to evolve to DG1111 by the routes of Fig. 3A, each route passing through three interior nodes. The rate of evolution between two nodes of Fig. 3A, say D0000 (*A*) and D1000 (*B*), is given by

(1) \begin{equation*} R = {N_A}u{f_B}, \end{equation*}

where *N_A_* is the number of individuals in node *A*, *u* is the mutation rate and ${f_B} = \ f ( {{N_A},\ s} ) = \frac{{1 - {e^{ - 2s}}}}{{1 - {e^{ - {N_A}s}}}}\ $ is the fixation probability of haplotype *B*, which has a selective advantage of *s* over haplotype *A*.

If the variant is neutral, *f_B_* = 1/*N_A_* and *R* = *u*. Hence, the ‘waiting time’ between two successive mutations (e.g. from 1000 to 1100) would be 1/*u*. Given the per base mutation rate of <10^–5^/bp/day [20–22], each step would take on average >10^5^ days. (Of course, if the mutation can fall on any site of the ∼30 kb genome, the waiting time would only be a few days.) With the four sites, the evolution from DG0000 to DG1111 would take much longer than one year.

With the slow rate of neutral evolution, speedier evolution at specific sites must be driven by natural selection. If both *N* and *s* are large, *R* can even be higher than 10^–2^ and it may take only 2–3 months to evolve from DG0000 to DG1111. However, the measurements of *s* (expressed as *D_w_* in Table 2) for the four partial haplotypes (DG0111, 1011, 1101 and 1110) are nowhere near the level that can reduce the evolutionary time to within a year. In fact, since these partial haplotypes all appear even less fit than the wild-type DG0000 (Table 2), the evolution of DG1111 from DG0000 would be blocked. This may imply that the evolution of DG0000 to DG1111 most likely happened in a genetic background different from that of the current DG0000; in other words, the evolution may have happened in an unknown haplotype background of a more distant past than shown in Fig. 2. In this background, at least one of the routes in Fig. 3A is not blocked.

### Testing the conventional one-beginning view—all strains spread initially from one place

The main message of Tables 1 and 2 is about the emergence of DG1111 in Europe as well as the non-emergence in China. The conventional view is that a (pre-DG1111) strain spread from China to Europe where it evolved into DG1111 (Fig. 2B). This view raises two related questions: (i) Which one is this pre-DG1111? (ii) How much time is available for this pre-DG1111 strain to evolve into DG1111 in Europe? (Here, we may also ask why DG1111 did not emerge in China and the simplest answer is the stochastic nature of evolution—not everything that could happen did happen.)

In answering the questions, we divide the evolution of the DG haplotypes in China into three stages in Fig. 2B (see also Table 1). In the Early stage before 21 January 2020, the haplotypes are entirely DG0000 (91/91). In the Middle stage between 21 January 2020 and 1 March 2020, there is no DG1111 haplotype yet but partial haplotypes (such as DG0001, DG1001 and DG1101) began to emerge albeit collectively accounting for only 12 out of 692 samples (1.7%). In the Late stage after 1 March 2020, DG1111 appeared and increased to 50% by the end of March. The increase happened with the inflow from abroad. In contrast, the DG1111 haplotype is present in *all* European and North American samples including the earliest collection dated 21 January 2020 (or even 1 January in an unusual Canadian sample, see Tables S4 and S5).

To answer the first question, we can conclude that the pre-DG1111 haplotype from Asia is not DG1111 itself, which was absent in China. The most common partial haplotype in China and Europe in the Middle stage is DG1101. However, as DG1101 appears at the same time in both continents and is far more common in Europe (∼20%) than in China (∼1%), DG1101 is unlikely to be the putative pre-DG1111 from China either. The other two partial haplotypes, DG-111 and DG1110, are both <0.2% in China in early February and have not been seen later (Table 1). They are even less likely to be the direct ancestor of DG1111 in Europe. (DG-111, in this interpretation, is most likely DG0111, as DG1111 would not have died out almost immediately.) In short, the import from China should be of only one possible haplotype—that of DG0000.

For a visual impression, genomic sequences of the three major haplotypes collected before 30 January 2020 are presented in Fig. 3B. The common DG0000, DG1111 and the partial haplotype DG1101 are shown. While the age should be in the order of DG0000, DG1101 and DG1111, the within-haplotype diversity appears the highest in the presumably youngest DG1111. The comparison implies that DG1111, when first detected, is already diverse, hinting at a period of mutation accumulation prior to the onset of the epidemics. This issue is pursued below.

The second question is about how much time is available for the evolution of DG1111 in Europe. The time span should be between the presumed arrival of DG0000 from Asia and the first appearance of DG1111 in Europe. We shall allow the earliest possible time for the arrival from Asia, which is 15 December 2019. The earliest possible time of the appearance of DG1111 is uncertain, but definitely no later than 10 February 2020. By that date, 7 of the 32 European samples are DG1111 and, 10 days later (20 February 2020), it is 18 of the 37 samples (Table 2). Nevertheless, the most interesting samples are those collected in January 2020, marked by the blue circled number in Fig. 2B (also see Table S6). As annotated in the legends, the sequence deposition in this period is unusually prone to revisions and retractions. Still, unless all these reports are false, the full DG1111 haplotype has been formed, likely in Europe, by the beginning of January 2020. Given the presence of DG1111 in small samples, often one out of one, the frequency may be quite high. Two recent reports on even earlier appearances of SARS-CoV-2 in Italy are discussed in the next section.

The distribution of DG1111 across continents in the beginning of the epidemics rejects the single-beginning scenario depicted in Fig. 2B whereby DG1111 has to evolve from DG0000 to DG1111 in an impossibly small time span. Furthermore, the earliest appearance of DG1111 is associated with very high nucleotide diversity as shown in Fig. 3B, indicating the real age of DG1111 as being much older than its first detection in humans. To conclude, if the spread from Asia to Europe has to be months before there was any sign of an impending epidemic, we would effectively be considering multiple beginnings of the epidemics.

### The multiple-beginning scenario—evidence from Europe

In discussing the beginning of an epidemic, we should note the distinction between the beginning and the origin. The place where SARS-CoV-2 originated is referred to as PL0. It has been suggested that PL0 is not the same as PL1, the first place that reports the impending epidemic [23,24]. To allow the multi-step adaptive shift from animal to human hosts, PL0 needs to possess several characteristics that are distinct from those conducive to the first epidemic [23]. The *beginning* of the epidemics is hence at PL1, which receives the infections directly from PL0 and spreads the virus to other places, collectively referred to as PL2s. It is often assumed that there is only one PL1 and it is in China, even though the theory supports multiple PL1s. This assumption will be tested here.

**Figure 4.**
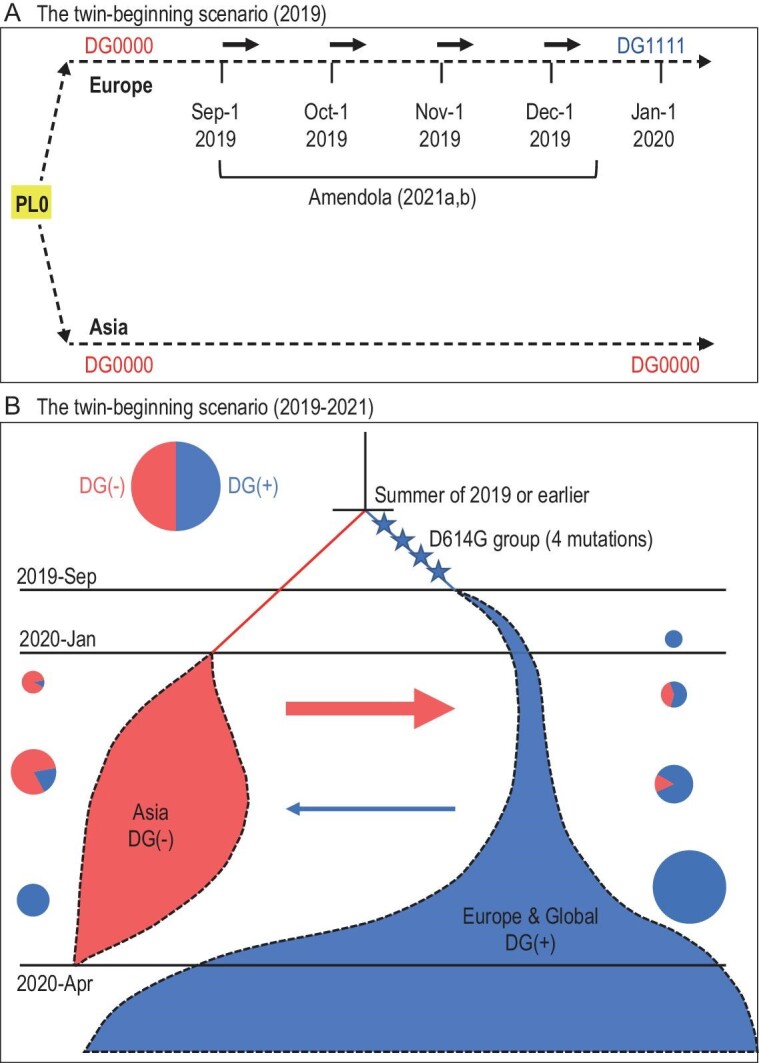
The twin-beginning scenario. (A) In this scenario, the split between the Asian and European lines occurred before September 2019. This scenario would allow more time for the evolution from DG0000 to DG1111 in Europe. The time period when SARS-CoV-2 was found in Italy is marked. It suggests that the evolution from DG0000 to DG1111 may have happened even earlier than indicated (see Amendola *et al*. 2021b). During the period, there is no parallel evolution, which would mean DG0000 evolving to DG1111 independently in both regions (see Discussion). Neither are there additional mutations, outside of the 4 DG group, that rise to high frequency in either Asia or Europe. (B) The evolution of SARS-CoV-2 from the beginning. All time points are the sampling dates reported. The Asian and European lineages may have coexisted but unnoticed before September 2019. By 1 October 2019, the DG group of mutations had already been assembled in Europe. In the subsequent period, from 1 October 2019 to 1 April 2020, the two lineages interact via gene flow (shown by the arrows) and competition. The frequency of the DG(+) vs. that of DG(−) is shown in the pie charts for both populations. DG(+) or DG(−) means ‘predominantly DG1111 or DG0000’, respectively. The size of the pie chart reflects the number of infections at that time. From 1 April 2020 onward, the global sweep of DG1111 mutations is nearly complete.

A parsimonious explanation for the emergence of DG1111 may be the twin-beginning scenario shown in Fig. 4A. In this scenario, the virus spread to both continents quite early, presumably from the yet unidentified PL0. With the independent beginning in Europe, the virus would have sufficient time to evolve the four DG mutations.

There have been many reports that SARS-CoV-2 was circulating in Europe (and perhaps in the USA) in late 2019 [25–32]. Among the most convincing studies is that of Amendola *et al*. [28]. In this report, a 4-year-old child has been shown, by PCR and DNA sequencing, to be infected with SARS-CoV-2 in November 2019 in northern Italy [28]. More recently, Amendola *et al*. (2021b) further show that SARS-CoV-2 was indeed circulating in the Lombardy region in September to December 2019 with 11 of the 44 suspected patients yielding SARS-CoV-2 sequences [32]. They further demonstrate that 6 out of the 11 patients have sequence reads covering at least one DG site. All six patients thus appear to have the DG group mutations when data are available. By early December 2019, the DG1111 haplotype had already been assembled in Europe but remained absent in Asia. If we consider the strong association among the DG group mutations, the first appearance of DG1111 in Europe could be as early as September 2019, a timeline implied in Amendola *et al*. (2021b) and adopted in Fig. 4B.

Figure 4B summarizes the evolution of the Asian and European lines of SARS-CoV-2 proposed in Fig. 2B. The two lineages compete in the spread much like the competition among sets of mutations depicted in Fig. 1. Although the Asian lineage has begun the spread slightly earlier, the main trunk of SARS-CoV-2 evolution is dominated by the European lineage (see the W0 wave of Fig. 1). Since April 2020, the Asian lineage has nearly disappeared from the epidemics. The period between late January and April 2020 is when the competition between the two lineages could be observed as shown by the pie charts of Fig. 4B. These pie charts illustrate the haplotype compositions in, as well as the viral exchanges between, Asia and Europe.

## DISCUSSION

As emphasized above, the *beginning* of the epidemics is at PL1. In theory, once the virus evolves into its epidemic form in PL0, it can spread simultaneously to multiple PL1s [23]. This study shows that Asia and Europe could both have PL1s within its boundary (designated by the red and blue colors in Fig. 4). However, only the PL1 that first reports the impending epidemic is identified as such and all other PL1s would be recognized as PL2. This is the caveat against the single-beginning view as the first one is perceived as the only one.

For COVID-19, the single-beginning view has been widely accepted but has never been tested. In this study, we provide the evidence from sequence evolution to reject this view. In brief, the full DG1111 haplotype emerged in Europe at about the same time as (or even earlier than) the arrival of Asian strains in Europe. Crucially, the Asian strains arriving in Europe must be of the DG0000 type because even partial haplotypes (such as DG1011) were not seen in China in the early samples.

The necessary condition for the W0 wave to rise and usher in the global pandemic is the assembly of the full DG1111 haplotype. Table 2 shows that DG1111 has a much higher fitness than the wild-type DG0000 whereas all other partial haplotypes are no better, and often worse, than DG0000. Our analysis shows that the Chinese haplotypes are far from the successful assembly of DG1111. Since the strains in China fail to evolve to DG1111 *in situ*, it is unlikely that a small cohort would succeed in this evolution immediately upon arrival in Europe.

It would thus appear that SARS-CoV-2 had been in Europe long enough to evolve DG1111 in separation from the viral evolution in Asia. Indeed, Amendola *et al*. (2021a, b) provide the data supporting this conjecture [28,32]. With the twin-beginnings, the place in China that takes the credit of being PL1 (or even PL0) is not even the one that dominates in the subsequent global spread; it is merely the first one to be out of the gate. The remaining issue is why the weaker DG0000 strain in China was noticed earlier than DG1111 became common in Europe. One simple explanation is the different *R*_0_ (basic reproduction number) valuers in different socio-behavioral settings in different regions (such as higher population density in urban markets of bigger cities). Moreover, early epidemics are highly stochastic even with the same *R*_0_ value. As shown by Ruan *et al*. (2021), the early dynamics are characterized by frequent failures and occasional successes of epidemics [23].

The crucial timeframe to see the beginning(s) is the few months before and after the onset of the epidemics. In this period, two concurrent lineages compete, ending with the complete replacement of the Asian variants by the European ones. The rise of the European DG1111 is the first wave of mutations in COVID-19. Indeed, waves of mutations appear rather common in epidemics. The 1918 flu is one earlier example [33,34] but COVID-19 has revealed this pattern with unprecedented clarity.

Finally, any attempt at interpreting the COVID-19 data of the first two months of 2020 as well as the last few weeks of 2019 has to heed the data accuracy in a chaotic time. Certain aspects of the data are much more robust than others. For example, the timing of data based on limited and localized samples can be inaccurate and potentially flawed. Conversely, relative frequencies of variants from multiple countries over an extended period of time are much more reliable. Such large-scale regional trends of the variant frequencies are far less likely to be affected by reporting errors. In determining the merits and limitations of studies of SARS-CoV-2’s evolution in the early 2020, these considerations must be factored in.

## MATERIALS AND METHODS

This section can be found in the Supplementary Material.

## DATA AVAILABILITY

All genomic data in this study are available, on registration, from GISAID (https://www.gisaid.org/).

## Supplementary Material

nwab223_Supplemental_Files
