# Supplementary material for: The twin-beginnings of COVID-19 in Asia and Europe—one prevails quickly
Source: Natl Sci Rev. 2021 Dec 11;9(4):nwab223. doi: 10.1093/nsr/nwab223 (PMC9046579; doi:10.1093/nsr/nwab223)
Supplement: nwab223_Supplemental_Files [file nwab223_supplemental_files.zip › Supplement.docx]

Supplementary Materials for

The twin-beginnings of COVID-19 in Asia and Europe – One prevails quickly

Yongsen Ruan^1^, Haijun Wen^1^, Mei Hou^1^, Ziwen He^1^, Xuemei Lu^2^, Yongbiao Xue^3^, Xionglei He^1^, Ya-Ping Zhang^2*^, Chung-I Wu^1, 3, 4*^

Correspondence to: [wzhongyi@mail.sysu.edu.cn](mailto:wzhongyi@mail.sysu.edu.cn), [ciwu@uchicago.edu](mailto:ciwu@uchicago.edu) (Chung-I Wu)

**This PDF file includes:**

Materials and Methods

Figs. S1 to S2

**Other Supplementary Materials for this manuscript include the following:**

Tables S1 to S7

Materials and Methods

**Virus sequences pre-processing**. We download 2,063,459 SARS-CoV-2 genomes from the GISAID database [1] as of July 5, 2021 with the following download options: (1) “complete”: genomes > 29,000nt; (2) “low coverage excel”: exclude viruses with >5% Ns (undefined base).We filtered out sequences without the collection date or with an implausible date (e.g. ‘1900-01-05’) and retained 1,853,355 genomes for downstream analysis. Note that the meta information (especially the collection date of viruses) of sequences could be changed or removed after several weeks (e.g., the 31 sequences collected on 2020-01-01 in Canada are currently not available). We mark these sequences as deposition irregularity.

**Sequence alignment, SNP calling and annotation**. We aligned these 1,853,355 genome sequences to the reference sequence (Wuhan-Hu-1 [2], GenBank: NC_045512, GISAID: EPI_ISL_402125) using MAFFT (--auto --keeplength) [3]. We used snp-sites (-v) [4] to identify SNPs and BCFtools (merge --force-samples -O v) [5] to merge the vcf files. Interestingly, although the reference genome size is only 29,903 nt, we found 67,650 SNP sites from these 1,853,355 genome sequences, indicating multiple substitutions at the same sites. We annotated the 67,650 SNP sites by ANNOVAR [6].

**Analysis of site frequencies in UK.** In this study, we track the variant frequency at each variable site (e.g., C🡪T). If we set the cutoff by ignoring variants that fail to reach 0.3 in frequency, there are usually fewer than 100 variants to keep track of. To observe the changes in site frequencies in a geographic region, we group the data into bins each covering a 10-day period. Only variants reaching the frequency cutoff of 0.3 at their peaks are retained. For the retained variants, we grouped them by pairwise Pearson coefficients with the cutoff of 0.9 (i.e., if the Pearson coefficient across the time span between two variants is equal or greater than 0.9, they would belong in the same group). With this cutoff, there are 5 major waves (W0 to W4) in UK (see Table S1, Fig 1A).

**Confirmative re-analysis of the DG group variants**. The sequences from GISAID database are mixed in quality and the meta information may be changed or even be removed as mentioned above. To update the meta information of the sequences, we re-downloaded the 56,508 genome sequences as of 2020-03-31 to see the early evolution of the 4 DG group variants (download date: 2021-08-16). Same as previous section, we removed sequences without complete date and remained 49,992 genome sequences of SARS-CoV-2 in human host. We then aligned these sequences to reference sequence (Wuhan-Hu-1) and extracted the four bases of the 4 DG group sites (241, 3037, 14408, and 23403) for each sequence (see Table S7). Because the number of sequences was small in some geographic regions, we separated the sequences by a 10-day (instead of 1-week) gap and then calculated the frequencies of the D614G 4-base haplotypes.

**Outgroup sequences for determining the ancestral state of site variants.** We downloaded 16 SARS-CoV-2 sequences from bats and pangolins. For comparison, we also downloaded 1 S lineage sequence, 1 L lineage and 12 earliest DG1111 sequences (i.e., TTTT in reference positions 241, 3037, 14408, 23403). We then aligned these 30 sequences (by MAFFT) with reference genome (Wuhan-Hu-1) and used Mega-X [7] to construct the maximum likelihood phylogenetic tree (Fig. S1). To determine the ancestral state of 4 DG group sites, we extracted the bases on the four sites (Fig. S2). The phylogenetic tree and the haplotype of the four sites suggests that the ancestral state of DG group sites in human host can be DG0000 (CCCA) or DG0100 (CTCA). The analyses are based on DG0000 being the ancestral state while the alternative yields a stronger conclusion (see the main text).

**References**

1. Elbe, S, Buckland-Merrett, G. Data, disease and diplomacy: GISAID's innovative contribution to global health. *Glob Chall*. 2017; **1**(1): 33-46.

2. Wu, F, Zhao, S, Yu, B*, et al.* A new coronavirus associated with human respiratory disease in China. *Nature*. 2020; **579**(7798): 265-9.

3. Katoh, K, Standley, DM. MAFFT multiple sequence alignment software version 7: improvements in performance and usability. *Mol Biol Evol*. 2013; **30**(4): 772-80.

4. Page, AJ, Taylor, B, Delaney, AJ*, et al.* SNP-sites: rapid efficient extraction of SNPs from multi-FASTA alignments. *Microb Genom*. 2016; **2**(4): e000056.

5. Li, H, Handsaker, B, Wysoker, A*, et al.* The Sequence Alignment/Map format and SAMtools. *Bioinformatics*. 2009; **25**(16): 2078-9.

6. Yang, H, Wang, K. Genomic variant annotation and prioritization with ANNOVAR and wANNOVAR. *Nat Protoc*. 2015; **10**(10): 1556-66.

7. Kumar, S, Stecher, G, Li, M*, et al.* MEGA X: Molecular Evolutionary Genetics Analysis across Computing Platforms. *Mol Biol Evol*. 2018; **35**(6): 1547-9.


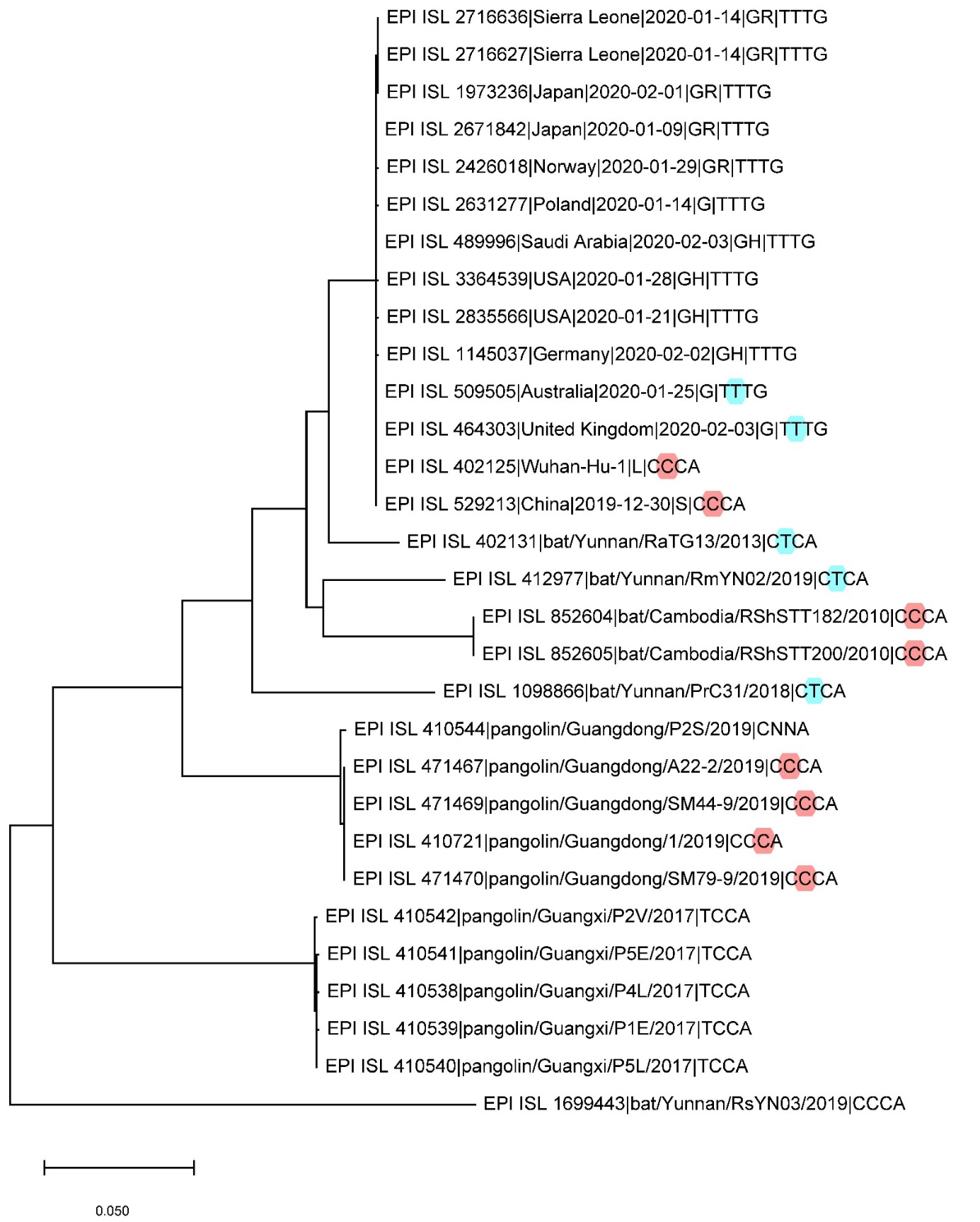


**Fig. S1**. The maximum likelihood phylogenetic tree of early SARS-CoV-2 sequences in human, bats, or pangolins.


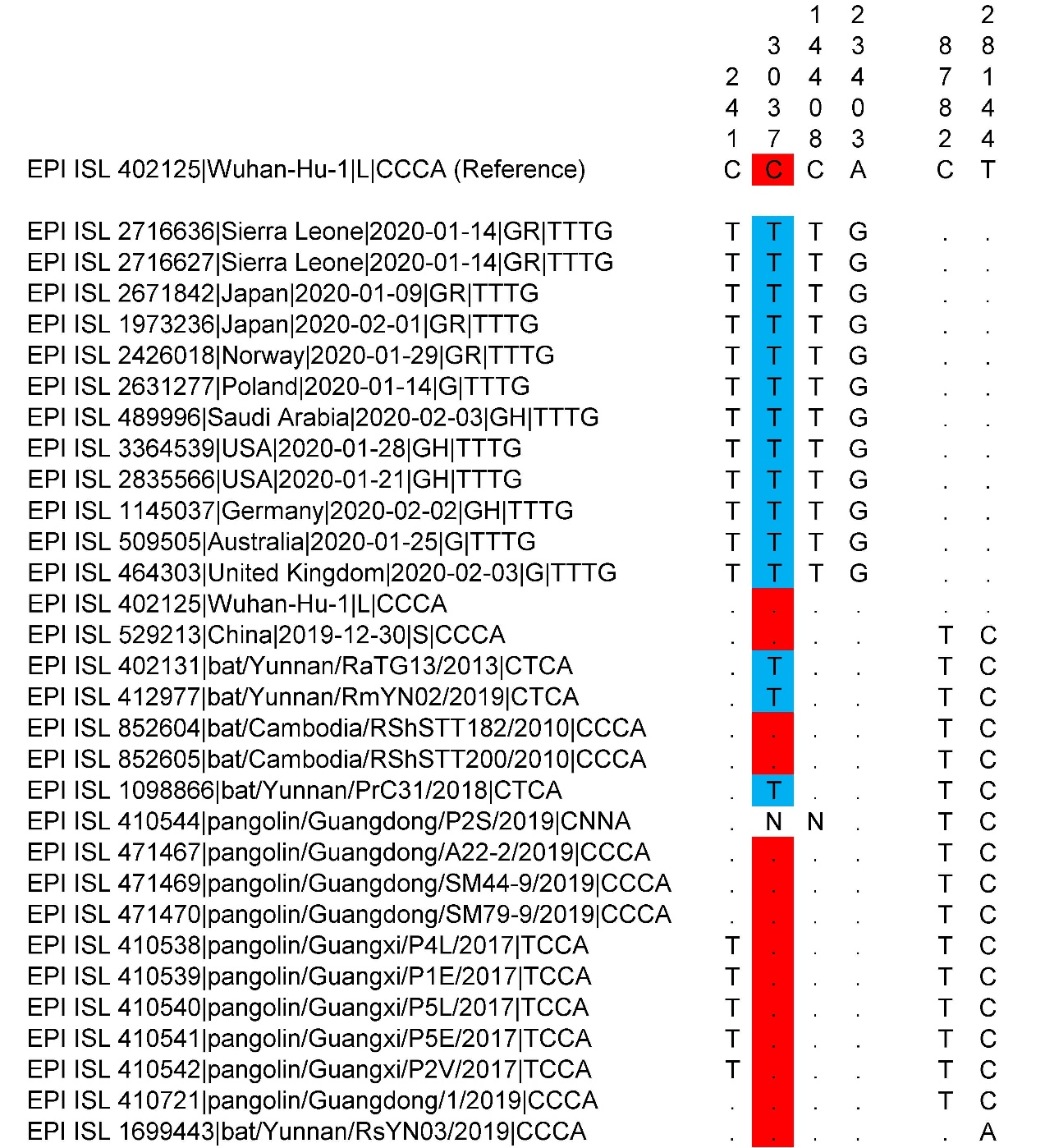


**Fig. S2**. The DG 4-base haplotype of early SARS-CoV-2 sequences in human, bats, or pangolins. All the sequences were aligned to reference genome (Wuhan-Hu-1). N represents any base; dot represent the same base as the reference genome.
